# Supplementary material for: Extreme Polaritonic Interactions in a Room‐Temperature Designable Sub‐Nanocavity Quantum Electrodynamic Platform
Source: Adv Sci (Weinh). 2026 May 19;13(44):e23335. doi: 10.1002/advs.202523335 (PMC13336004; doi:10.1002/advs.202523335)
Supplement: Supplementary file 1 [file ADVS-13-e23335-s001.pdf]

Supplementary Materials for Extreme polaritonic interactions in a room-temperature designable sub-nanocavity quantum electrodynamic platform

Huatian Hu,[†,‡] Xin Shu,[†] Zhiwei Hu,[¶] Di Zheng,[§] Ruiqian Zhang,[§] Ximin Cui,[§] Wei Dai,[‖] Xiang Lan,[⊥] Xiaobo Han,[*,†] Wen Chen,[*,¶] and Hongxing Xu[*,#,¶]

†*Hubei Key Laboratory of Optical Information and Pattern Recognition, Wuhan Institute of Technology, Wuhan 430205, China*

‡*Istituto Italiano di Tecnologia, Center for Biomolecular Nanotechnologies, Via Barsanti 14, 73010 Arnesano, Italy*

¶*State Key Laboratory of Precision Spectroscopy, East China Normal University, 200241 Shanghai, China*

§*State Key Laboratory of Radio Frequency Heterogeneous Integration, College of Electronics and Information Engineering, Shenzhen University, 518060 Shenzhen, China*

‖*School of Physics and Technology, Wuhan University, Wuhan 430072, China*

⊥*State Key Laboratory of Advanced Fiber Materials, Center for Advanced Low-dimension Materials, College of Materials Science and Engineering, Donghua University, Shanghai, China*

#*Institute of Laser Manufacturing, Henan Academy of Sciences, Zhengzhou, China.*

E-mail: hanxiaobo@wit.edu.cn; wchen@lps.ecnu.edu.cn; hxxu@hnas.ac.cn

Contents

S1 Methods **S3**

S1.1 Numerical simulations. S3

S1.2 Fabrication process of NPcoM sub-nanocavity. S3

S1.3 Nanoparticle Statistical Analysis. S4

S1.4 Dark-field scattering and PL spectroscopy. S7

S1.5 Uncoupled PL background quenching S8

S2 Fitting of anti-crossing curves. **S9**

S3 Lorentzian fitting of the plasmonic and excitonic linewidths. **S10**

S4 Fitting of scattering spectra by the coupled-oscillator model. **S11**

S5 Fabrication process of NPoM nanocavity as a reference sample. **S12**

S6 Comparison of the energy of the PL peaks from bare TMDC and hybrid systems **S13**

S7 Quenched PL of bare MoS_2 and PL spectrum of hybrid NPcoM **S14**

S8 Spatial variation of mode volume, LDOS and antenna efficiency **S15**

References **S19**

S1 Methods

S1.1 Numerical simulations.

Simulations were performed with the FEM package COMSOL Multiphysics 6.2. Two types of computation were implemented: (1) For the mode volumes in the main text Fig. 1 and the vacuum-field supported coupling strength in Fig. 5, we used quasinormal mode analysis and normalization as introduced in *MAN* package.[1] (2) For the field enhancement and the scattering spectrum under local and nonlocal[2] approximations in the main text Fig. 1, we used a p-polarized oblique incident plane wave to simulate experimental conditions. Due to the symmetry of the system, a 2.5D method was considered.[2] For both simulations, perfectly matched layers were used as boundary conditions to absorb the propagating waves in the free space. The permittivity of gold was taken from Olmon et.al. The refractive index of dielectric spacer was taken as 1.5.

S1.2 Fabrication process of NPcoM sub-nanocavity.

Firstly, an 80-nm-thick Au film was deposited on a silicon substrate by electron-beam evaporation, using a 5-nm chromium layer as an adhesion promoter. Subsequently, a self-assembled single layer of Au nanoseeds was transferred onto the Au film. For the self-assembly process (Fig. S1a1), 2 mL of 0.1 mM tetrabutylammonium nitrate was mixed with 1.2 mL of dichloromethane in a 5 mL centrifuge tube. Subsequently, a nanoseed solution was added, and the mixture was vigorously shaken for 30 s (Fig. S1a2). After allowing the mixture to stand for 2 minutes, 500 μL of n-hexane was carefully introduced along the tube wall. This step induced the migration of nanoseeds to the interface between the aqueous and organic phases, forming a densely packed single layer (Fig. S1a3). Excess n-hexane was removed without disrupting the nanoseed assembly.[3] The Au/Si substrate was then immersed into the solution, and the self-assembled nanoseed layer was transferred onto the Au film via slow withdrawal from the air/water interface, as shown in Fig. S1b. Next, 100 nm Au

nanoparticles were drop-cast onto the modified substrate. After 2 minutes of incubation to ensure sufficient surface attachment, the sample was rinsed with deionized water to remove unbound particles and dried under nitrogen flow. For transfer monolayer TMDCs, MoS_2 flakes grown on sapphire via CVD were transferred to packed nanoseeds/Au films using a PDMS-assisted method,[4] with careful handling to prevent dust contamination.

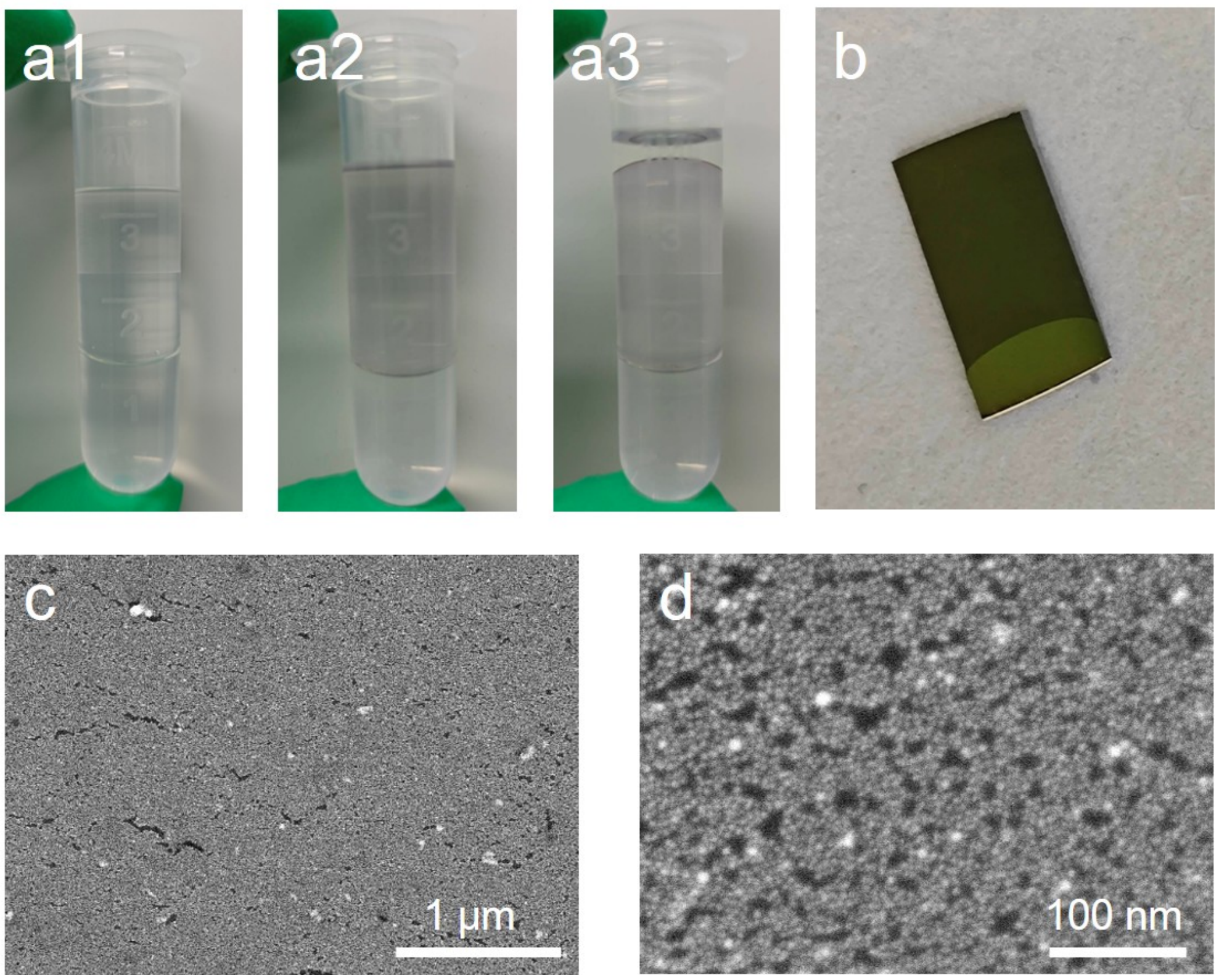

Figure S1: (**a1-3**) Pictures for the self-assembly process. (**b**) Self-assembly of Au nanoseeds on Au film. (**c-d**) SEM characterization of the closely packed nanoseeds on Au films at different magnifications.

S1.3 Nanoparticle Statistical Analysis.

Nanoparticle statistics were analyzed from both TEM and SEM images using home-developed MATLAB programs. For TEM images, a circular Hough transform was applied to identify nearly circular nanoparticles based on grayscale contrast (main text Fig. 2b, and Fig. S3). The average particle diameter ($\bar{D}$) was extracted from high-resolution TEM images. For SEM

image analysis, a combined method of object boundary tracing and circular Hough transform was employed. First, unassembled regions were excluded by tracing object boundaries to isolate the assembled area (A_{assm}) (Fig. S2a). Within these assembled regions, the circular Hough transform was used to determine the particle count (N) (Fig. S2b). Using the average particle size obtained from the TEM analysis, the coverage rate (C) was calculated as:

$$C = \frac{N\pi(\bar{D}/2)^2}{A_{\text{assm}}}.$$

Assuming a square lattice arrangement of nanoparticles, the effective gap (G_{eff}) was derived as:

$$G_{\text{eff}} = \sqrt{\frac{\pi(\bar{D}/2)^2}{C}} - \bar{D}.$$

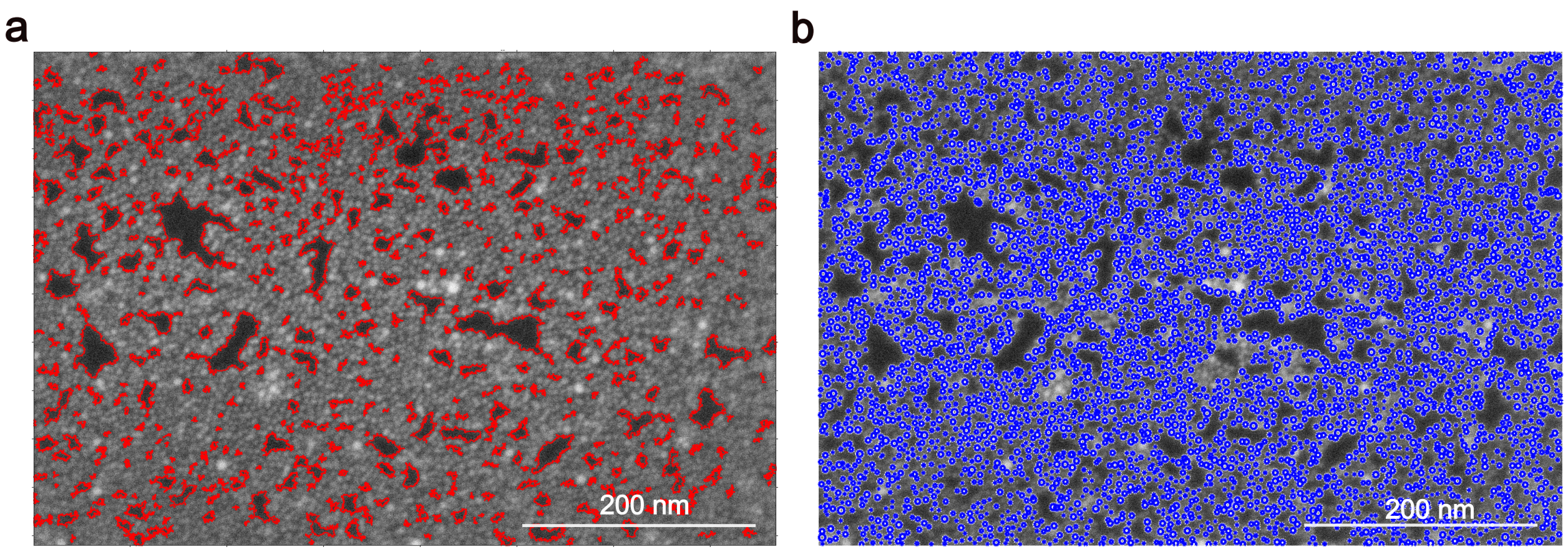

Figure S2: (**a**) Using object boundary tracing to exclude unassembled regions in the SEM image. (**b**) Using the circular Hough transform to count nanoparticles.

To quantify how many nanoseeds can contribute to the sub-nanocavity hotspots and demonstrate the origin of device-to-device variability, a statistical analysis of nanoseed distribution within the nanoparticle-on-mirror nanocavity was performed (Fig. S3). The analysis was based on the TEM image shown in Fig. S3a, which was also used in the main text (Figs. 2b,c) for particle size and density characterization. The bottom facet of a 100 nm nanoparticle was approximated as a circular region with diameters of 20, 25, 30, and 40 nm.

A total of 3732 nanoseeds were identified in the TEM image. For each nanoseed, a circular

sampling window centered at its position was constructed, with the diameter corresponding to the assumed facet size (20–40 nm), resulting in 3732 sampling windows. Within each window, the number of nanoseeds was counted to estimate the local distribution of sub-nanocavity hotspots (Figs. S3b–d). A nanoseed was included in the count only if its center was located within the circular window. Nanoparticles at the edges of the TEM image were excluded. This sampling approach captures local density fluctuations of nanoseeds within realistic cavity footprints.

For a 20 nm facet, the average number of nanoseeds is ∼7.7 with a standard deviation of ∼1.4. For 25, 30, and 40 nm facets, the averages are ∼13.0, ∼17.9, and ∼31.8, with standard deviations of ∼2.0, ∼2.4, and ∼3.7, respectively. These statistical results provide a quantitative basis for the observed variations in Rabi splitting and PL enhancement across devices.

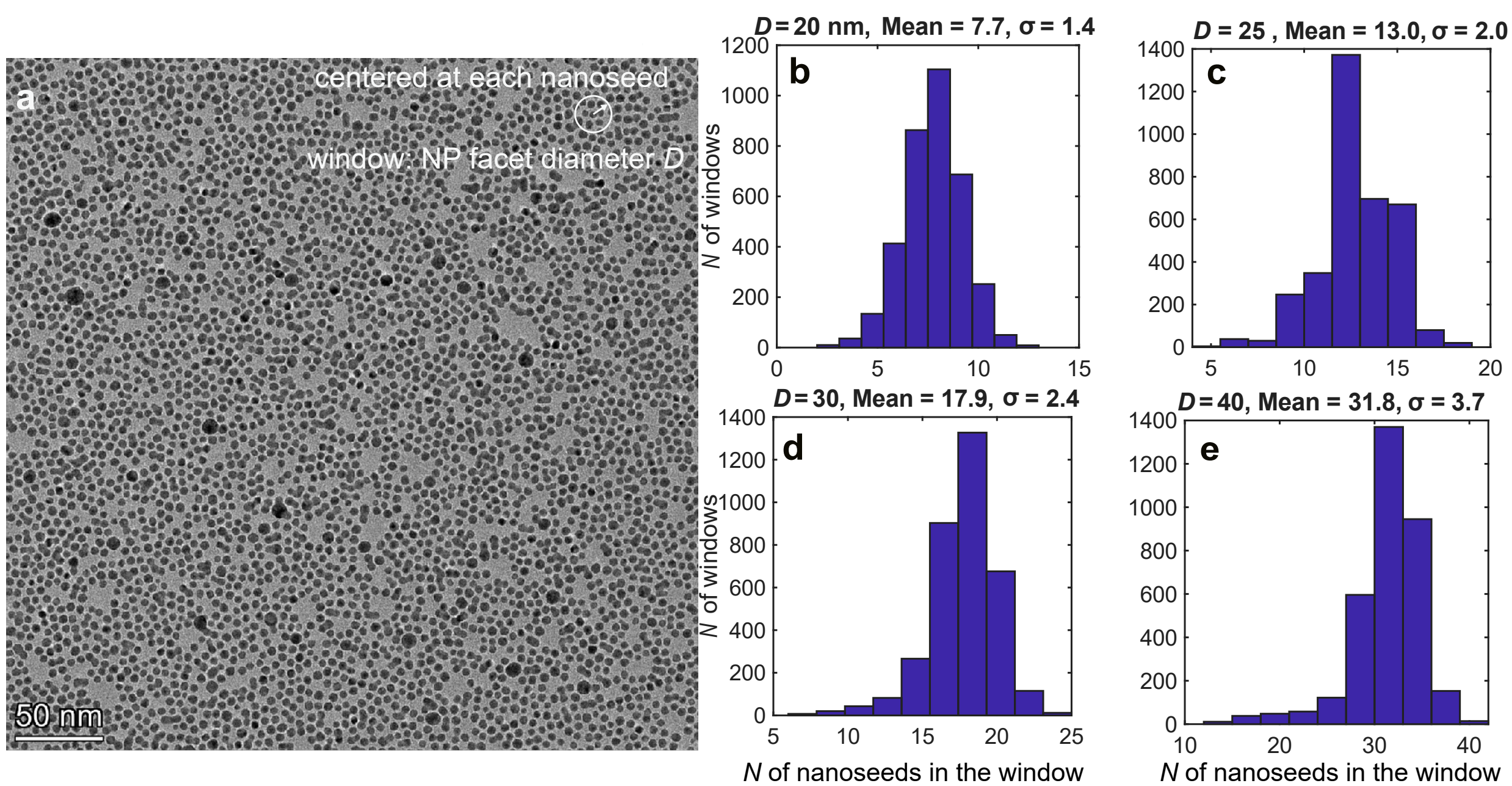

Figure S3: (**a**) Full TEM image of closely assembled nanoseeds from which the statistics (main text Fig. 2c) were performed. From (**a**), a statistical coverage analysis of a (**b**) 20 nm, (**c**) 25 nm, (**d**) 30 nm, and (**e**) 40 nm facets are performed to count how many nanoseeds can be sandwiched under the bottom facet of the NPoM nanocavity.

S1.4 Dark-field scattering and PL spectroscopy.

The optical characterization was performed using a dark-field microscopy system (Olympus BX53) equipped with a 100 W halogen lamp for broadband illumination. The sample was excited through a dark-field objective (Olympus MPLFLN 100×, NA = 0.9), and the scattered light was collected by the same objective. A lens focused the scattered signal onto the image plane, where a 100 μm pinhole was placed to spatially select individual nanoparticles. The filtered light was then directed through another lens to either a fiber-coupled spectrometer (Andor 303i) or a CCD camera (Qimaging QICAM B series) for spectral or imaging analysis, respectively. All raw scattering spectra were normalized by dividing the sample signal by the reference spectrum obtained from a standard white plate under identical measurement conditions. For PL characterization, the samples were excited using a 473 nm continuous-wave (CW) laser delivered through the same 100× objective (NA = 0.9). The focused laser spot size was precisely determined to be 1 μm in diameter using an improved knife-edge technique (Fig. S4). The excitation power was fixed at 700 μW. The identical excitation conditions were maintained for all measurements, including both NPCoM and NPoM nanostructures. For bare MoS_2, the regions without 100-nm Au particles near the NPCoM or NPoM structures were excited under identical power conditions.

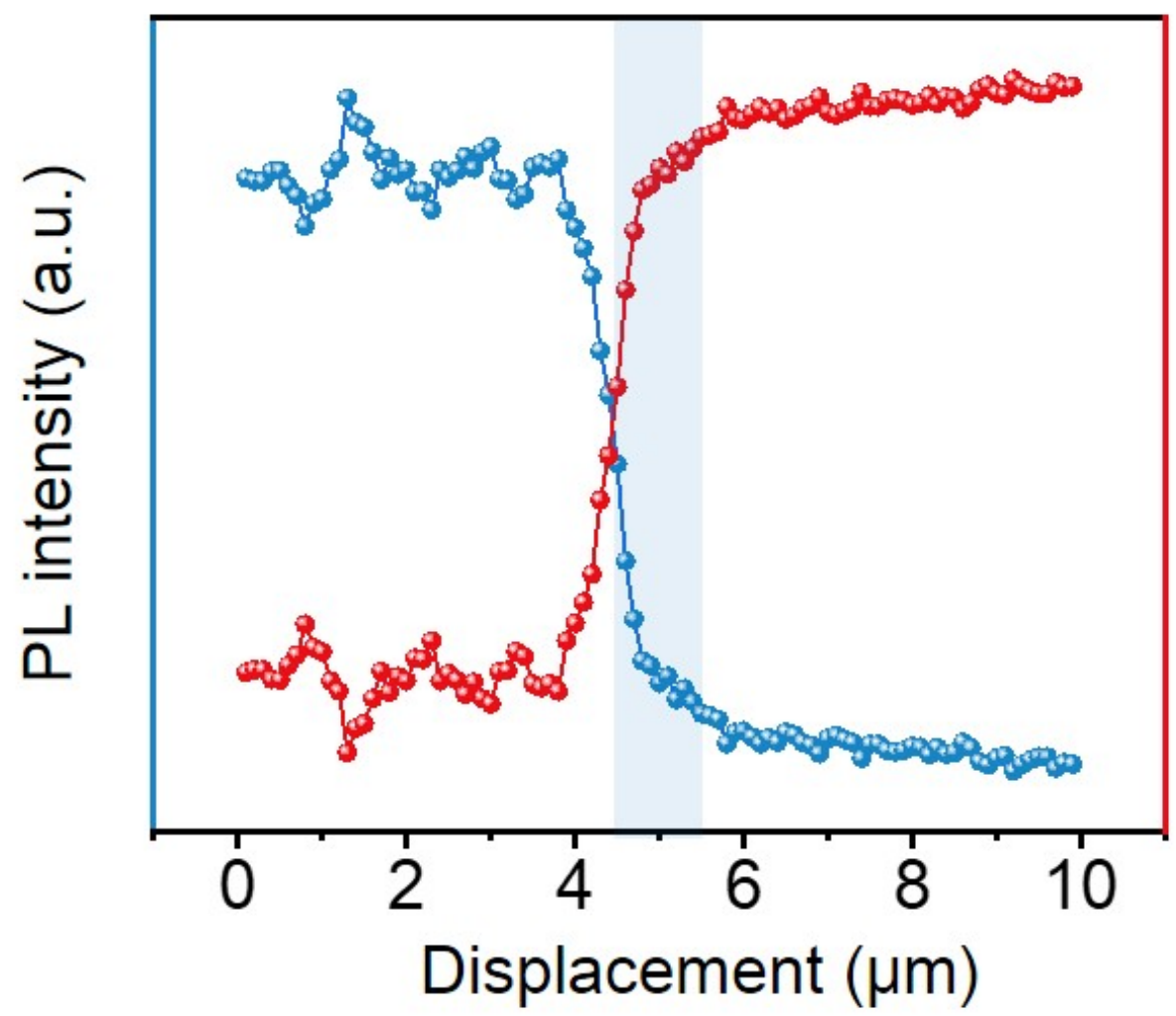

Figure S4: Laser spot size characterization via knife-edge scanning of monolayer MoS_2. A triangular MoS_2 flake was precisely translated across a fixed laser spot using a piezoelectric stage. The flake edge serves as a sharp knife-edge for beam profiling. PL intensity profile showing the transition when scanning across the flake edge. The 50%-100% intensity transition width of 1 μm (shaded region) corresponds to the laser spot diameter. The blue points represent the mirror-symmetric counterparts of the red points.

S1.5 Uncoupled PL background quenching

To quench the background signal from the sample, Au clusters were deposited by electron beam evaporation. The deposition was performed at a rate of 0.2 Å/s with a power setting of 8.5% of the maximum power, yielding Au clusters with an average diameter of ∼3 nm. These ultrasmall Au clusters effectively quenched the background signal while preserving the intrinsic properties of the sample surface. The evaporation was carried out under high vacuum to ensure Au clusters uniformity and minimize contamination.

S2 Fitting of anti-crossing curves.

By diagonalizing the Hamiltonian of the cQED system, the eigenvalues of the hybrid modes were obtained with the complete solutions expressed as:

$$E_{\mathrm{UP}} = \frac{S}{3} + A + \frac{Y}{A}$$
$$E_{\mathrm{MP}} = \frac{S}{3} - \frac{1}{2}\left(A + \frac{Y}{A}\right) - \frac{i\sqrt{3}}{2}\left(A - \frac{Y}{A}\right)$$
$$E_{\mathrm{LP}} = \frac{S}{3} - \frac{1}{2}\left(A + \frac{Y}{A}\right) + \frac{i\sqrt{3}}{2}\left(A - \frac{Y}{A}\right)$$

where the intermediate variables are defined as:

$$S = \hbar(\tilde{\omega}_{\mathrm{c}} + \tilde{\omega}_{\mathrm{A}} + \tilde{\omega}_{\mathrm{B}})$$
$$R = \frac{\hbar\tilde{\omega}_{\mathrm{B}} g_1^2}{2} + \frac{\hbar\tilde{\omega}_{\mathrm{A}} g_2^2}{2} - \frac{S^3}{27} + \frac{S \cdot [-g_1^2 - g_2^2 + \hbar^2(\tilde{\omega}_{\mathrm{A}}\tilde{\omega}_{\mathrm{B}} + \tilde{\omega}_{\mathrm{A}}\tilde{\omega}_{\mathrm{c}} + \tilde{\omega}_{\mathrm{B}}\tilde{\omega}_{\mathrm{c}})]}{6} - \frac{\hbar^3\tilde{\omega}_{\mathrm{c}}\tilde{\omega}_{\mathrm{A}}\tilde{\omega}_{\mathrm{B}}}{2}$$
$$Y = \frac{S^2}{9} + \frac{g_1^2 + g_2^2}{3} - \frac{\hbar^2(\tilde{\omega}_{\mathrm{A}}\tilde{\omega}_{\mathrm{B}} + \tilde{\omega}_{\mathrm{A}}\tilde{\omega}_{\mathrm{c}} + \tilde{\omega}_{\mathrm{B}}\tilde{\omega}_{\mathrm{c})}}{3}$$
$$\Delta = R^2 - Y^3$$
$$A = \sqrt[3]{\sqrt{\Delta} - R}$$

S3 Lorentzian fitting of the plasmonic and excitonic linewidths.

As exemplified in Fig. S5a, the plasmon linewidth Γ_c is obtained from Lorentzian fitting of the dark-field scattering spectra of the NPcoM cavity mode by replacing the excitonic material by 1 nm Al_2O_3 layer. We took the average linewidth of 20 NPcoMs to obtain a mean Γ_c = 205 meV (the device to device variation is shown in Fig. S5b). As shown in Fig. S5c, the excitonic linewidth Γ_{ex} is extracted from the absorption spectra of the bare monolayer MoS_2 on the realistic substrate of NPcoM (without the nanoparticles on top).

The extracted Γ_{ex} inherently contains both homogeneous and inhomogeneous contributions, arising from spatial variations in local dielectric environment, strain, and defects. Experimentally measured spectra already incorporates inhomogeneous broadening, preventing direct separation of homogeneous and inhomogeneous contributions, it provides a realistic yet conservative estimate of the excitonic dissipation in the system. The presence of inhomogeneous broadening tends to increase the apparent linewidth and thus makes the strong coupling criterion more strict.

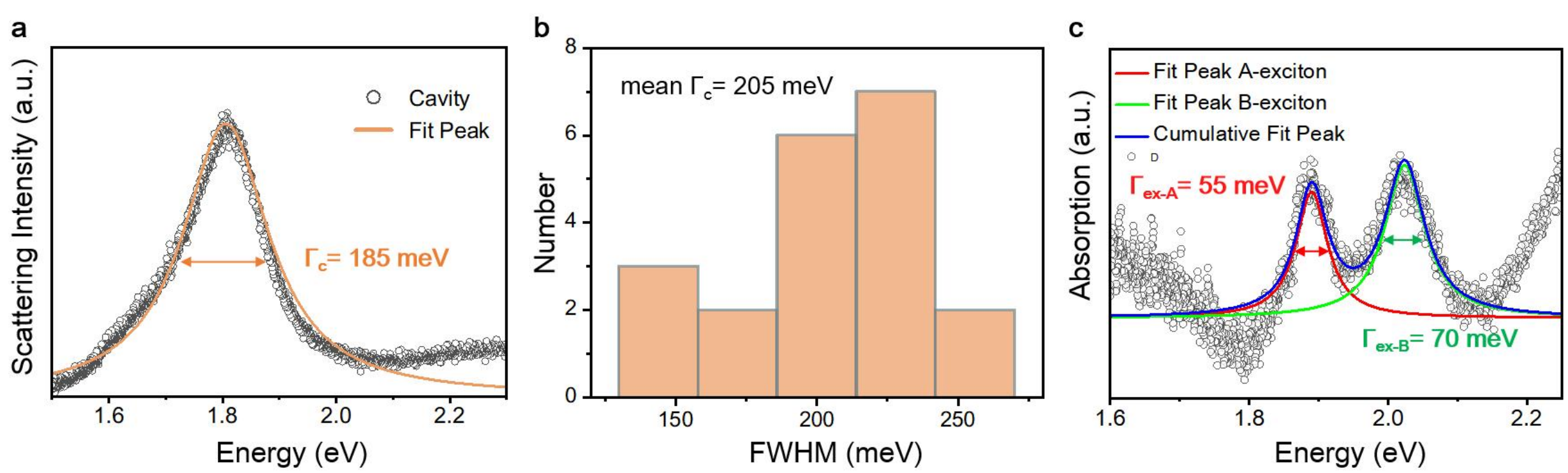

Figure S5: **(a)** Lorentzian fitting of the scattering spectra of NPcoM nanocavity with Γ_c =185 meV, **(b)** Statistical of Γ_c from 20 nanocavities, and **(c)** Lorentzian fitting of the absorption of monolayer MoS_2 with Γ_{ex-A}=55 meV and Γ_{ex-B}=70 meV.

S4 Fitting of scattering spectra by the coupled-oscillator model.

According to the complete and simplified coupled oscillator models (COM) for strong coupling established in previous work,[5] each scattering spectrum can be fitted to extract the coupling parameters. The data list and statistics are shown in Table S1 and Fig. S6 .

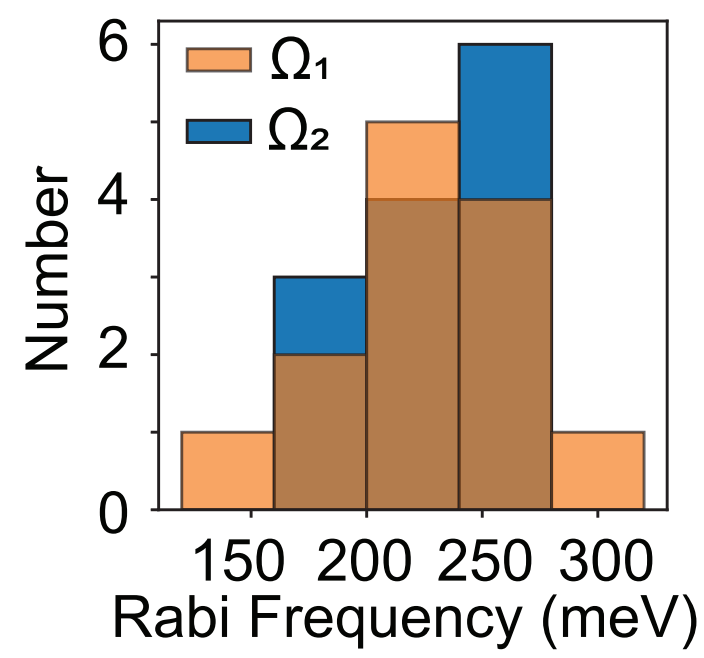

Figure S6: Rabi splittings extracted from single sub-nanocavity polaritons using a coupled-oscillator model, showing device-to-device variations in coupling strength. The average coupling strength is approximately 200 meV, consistent with the fitting in main text Fig. 3**(b)**.

Table S1: List of data corresponding to Figure S6

NO.	Ω_1	Ω_2
1	229.8	172.4
2	217.4	202.6
3	200.6	260.0
4	180.0	251.8
5	248.0	248.8
6	260.0	173.0
7	200.0	260.0
8	239.6	207.4
9	245.4	220.0
10	258.6	200.8
11	280.0	183.6
12	156.2	255.8
13	183.0	260.0

S5 Fabrication process of NPoM nanocavity as a reference sample.

Similar to the fabrication process of NPcoM sub-nanocavity, Au films were prepared on Si substrates first. A 5-nm Al_2O_3 was then grown by atomic layer deposition (ALD) as the spacer layer. Monolayer MoS_2 was subsequently transferred onto this substrate surface, followed by the controlled dispersion of 100-nm Au nanoparticles to complete the assembly. Figure S7a displays the schematic representation of the NPoM nanocavity geometry. The measured scattering spectra and anti-crossing curves are shown in Fig. S7c and S7b, with a Rabi splitting of 130 meV.

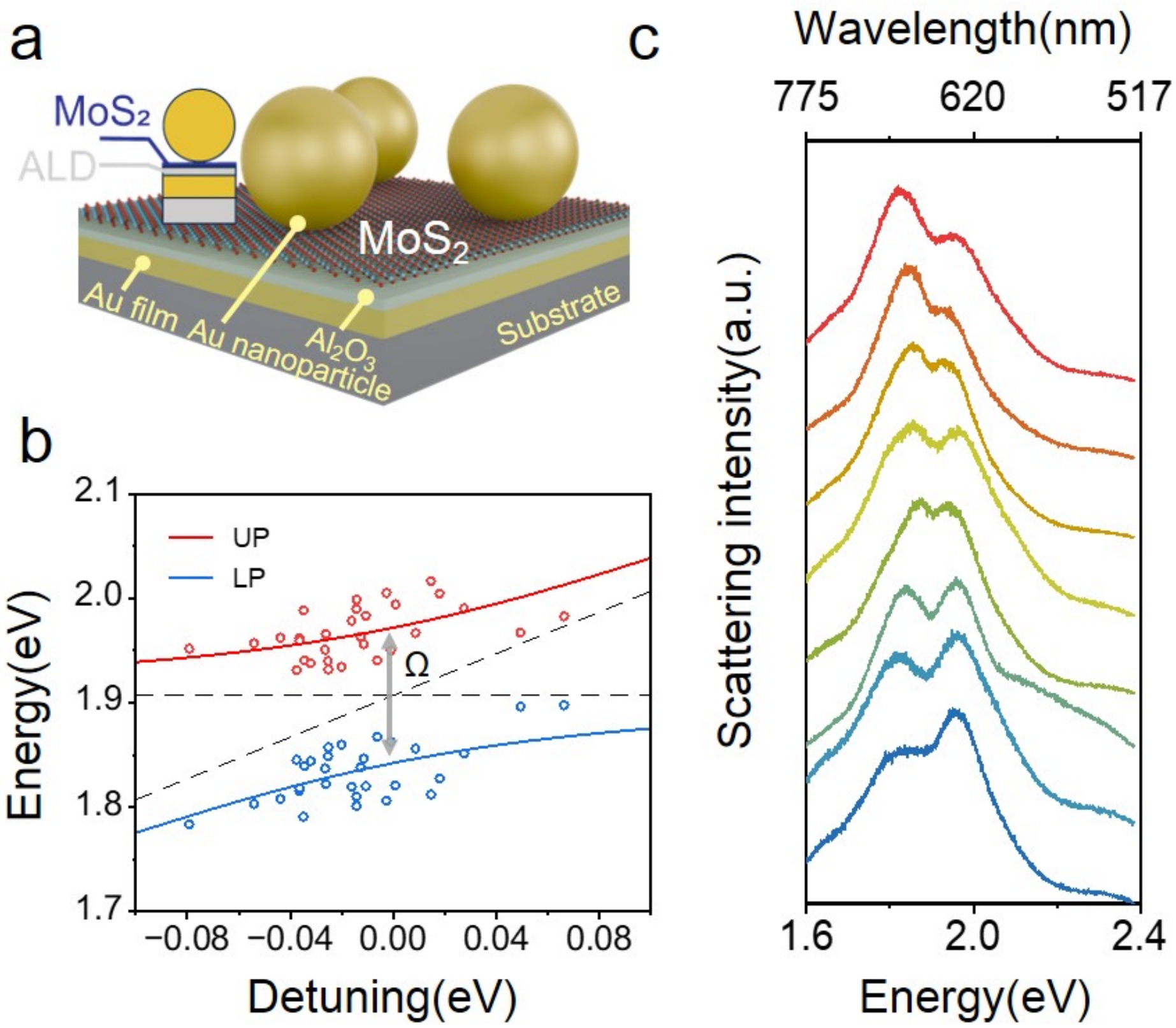

Figure S7: (**a**) Three-dimensional schematic of the NPoM nanostructure, with inset showing its two-dimensional cross-sectional view. (**b**) Anti-crossing dispersion featuring a Rabi splitting (Ω = 130 meV). (**c**) Scattering spectra of the NPoM cavities coupled with monolayer MoS_2.

S6 Comparison of the energy of the PL peaks from bare TMDC and hybrid systems

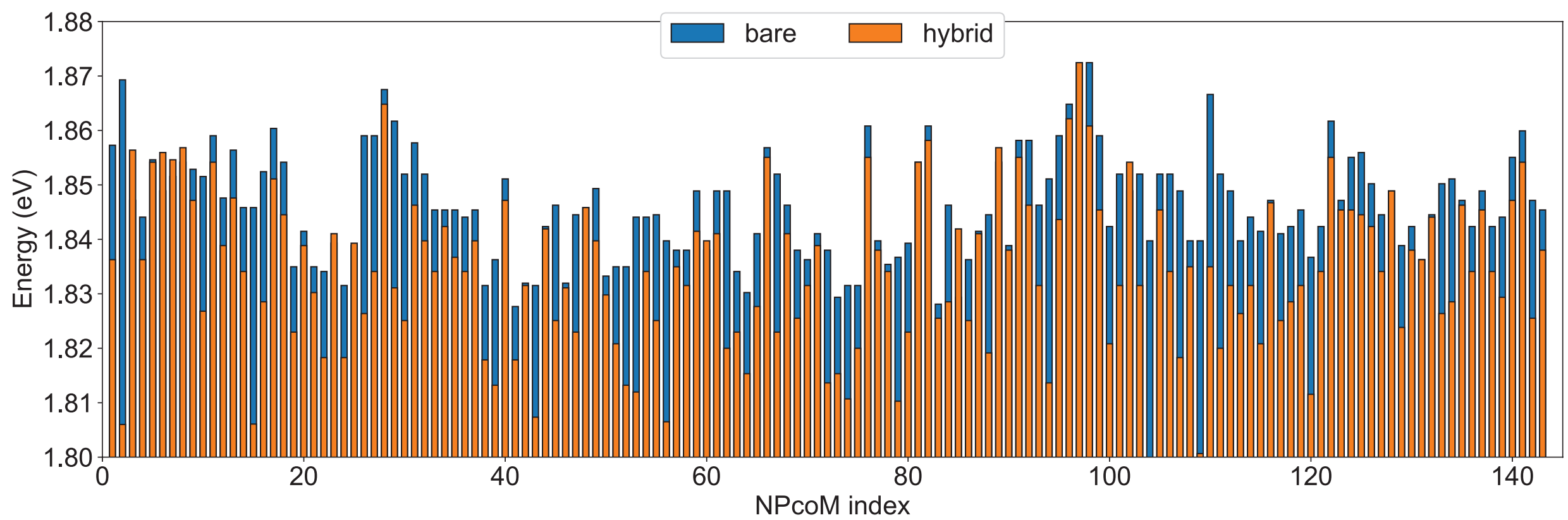

Figure S8: Comparison of the energy of the PL peaks from bare monolayer MoS_2 (blue) and the hybrid NPcoM-MoS_2 hybrid systems (orange). We measured more than 140 nanoparticles (indexed at the x-axis). The energy of the bare MoS_2 (blue) is generally higher than the hybrid systems, manifesting that the PL from the hybrid systems is contributed by the lower-polaritonic branch, instead of barely uncoupled excitons (which should stay at the energy marked by the blue boxes). It proves that we are investigating the polaritonic PL.

S7 Quenched PL of bare MoS_2 and PL spectrum of hybrid NPcoM

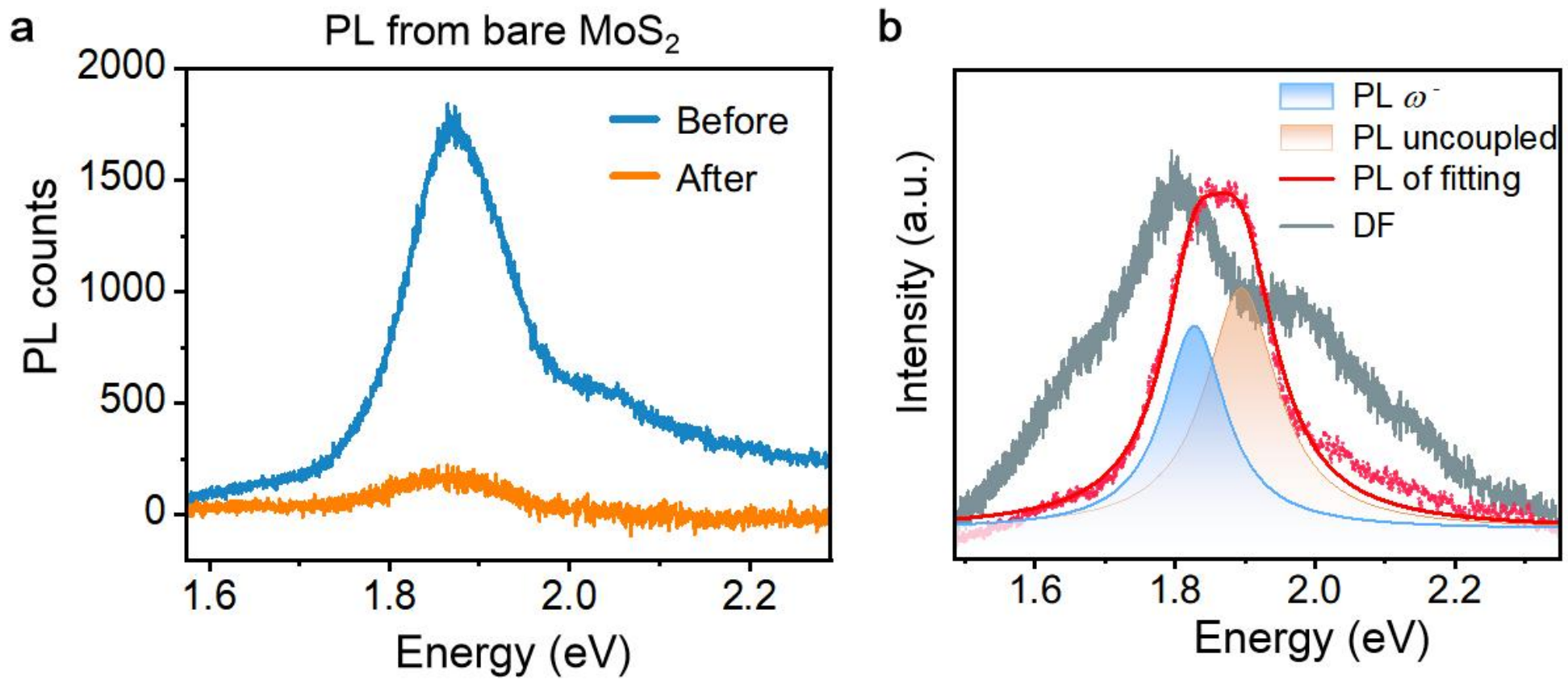

Figure S9: **(a)** Typical PL spectra of bare MoS_2 before and after evaporated small nanoparticles. (**b**) PL spectrum of quenched hybrid NPcoM nanocavity. The splitting of the DF scattering from the same NPcoM (gray) is plotted as a reference.

S8 Spatial variation of mode volume, LDOS and antenna efficiency

The mode volume ($V_m \sim 55$ nm^3) reported in this work corresponds to the peak-localized value at the position of maximum field confinement, following the standard definition in nanophotonics. Due to the highly confined nature of the sub-nanocavity mode, the effective V_m is strongly dependent on the spatial overlap between the emitter and the hotspot. Numerical mapping and contour plot of $1/V_m$ (Fig. S10) shows that a lateral displacement of only a few nanometers can increase the effective mode volume by more than one order of magnitude, indicating that spatial variation plays a critical role in determining the observed coupling strength and emission enhancement.

To assess experimental consistency, we estimate the Purcell factor based on the simulated V_m, according to,

$$F_p = \frac{3}{4\pi^2}\left(\frac{\lambda}{n}\right)^3 \frac{Q}{V_m}. \tag{1}$$

Using representative parameters ($\lambda \sim 660$ nm, $Q \sim 8$, $n \sim 1.5$, $V_m \sim 55$ nm^3), we obtain a total Purcell factor of $\sim 9.4\times10^5$. After applying a nonlocal correction factor of ~ 0.81 (estimated from the field enhancement with and without nonlocal correction), this yields an effective Purcell factor of $\sim 7.6\times10^5$. We note that this value corresponds to the total decay rate enhancement and should be further weighted by the antenna efficiency (Fig. S10a) to estimate the observable PL enhancement. This results in an upper-bound PL enhancement of $\sim 4.6\times10^4$, which is consistent in order of magnitude with the strongest experimentally observed events (Fig. 3f).

Due to the strong spatial localization of the sub-nanocavity mode, the effective mode volume and corresponding Purcell enhancement are highly sensitive to the emitter position and nanoseed configuration. As illustrated in Fig. S10c, a lateral displacement of ~ 3 nm from the hotspot can increase the mode volume by a factor of up to ~ 37.5, corresponding to a similar reduction in the Purcell factor. This implies that the PL enhancement from a single

hotspot can decrease to the order of $\sim 10^3$ under non-optimal spatial overlap. Considering that typical NPcoM structures exhibit PL enhancement on the order of $\sim 5 \times 10^3$, this suggests that multiple nanoseeds (on the order of several units) contribute collectively to the observed emission. While this estimation is based on simplified assumptions, it captures the key trend that governs the experimentally observed PL response.

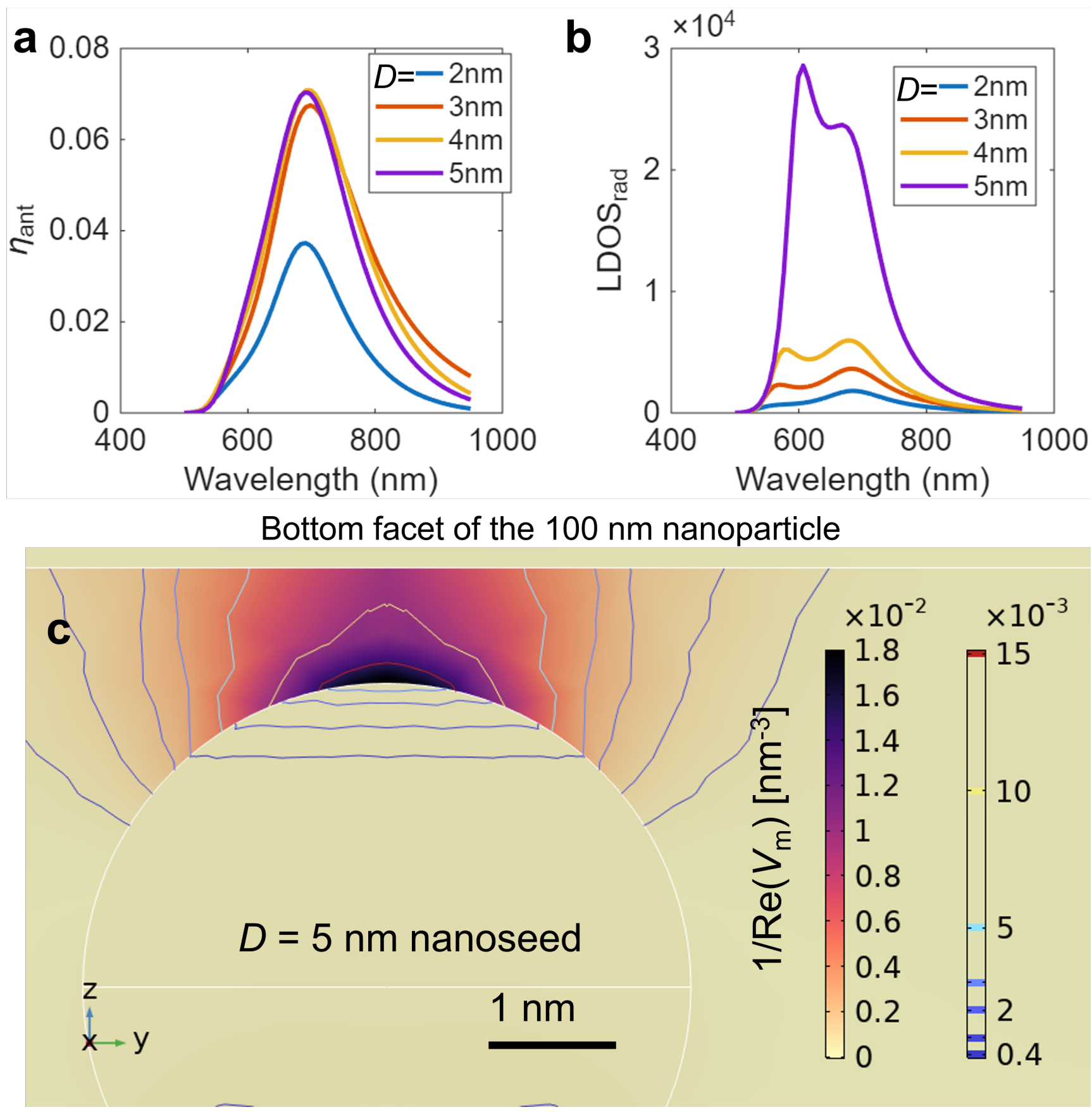

Figure S10: **(a)** The antenna efficiency (quantum efficiency) and **(b)** radiative local density state (LDOS$_{rad}$) of the NPcoM sub-nanocavity with a nanoseed diameter from 2 to 5 nm. The variables are defined as $\eta_{ant} = P_{rad}/P_{tot}$. P_{tot} is the total power emitted by the dipole, obtained by integrating the Poynting vector over a closed surface enclosing the dipole in the near field, and P_{rad} is the radiative power collected in the far field within the acceptance cone of the objective lens. **(c)** Contour of the mode volume $1/V_m$ showing that the sub-nanocavity presents a very localized field. A slight deviation from the center to the side of the nanoseed can lead to a 37.5 times larger mode volume (15/0.4) shown by the levels in the contour map.

Table S2: Strong coupling between QEs and different plasmonic nanostructures.

**Plasmonic nanostructures**	γ [meV]	κ [meV]	Ω [meV]	$\Omega/(\gamma+\kappa)$	**Ref.**
Au nanospheres on Au film	55	205	208	0.8	This work
Au nanohole array	28	36	60	0.9375	[6]
Chirped Ag nanoproove array	40	240	54	0.1928	[7]
Au nanoproove array	–	–	65	–	[8]
Ag coated monolayer of silica spheres	–	–	160	–	[9]
Ag nanorod array	28, 25	54, 60	47, 52	0.5732, 0.6118	[10]
Ag nanorods	43–70	50–190	41–133	0.2278–0.7597	[11–18]
Au@Ag nanocuboid	42–95	128–212	36.2–107.4	0.1261–0.6318	[19,20]
Au bi-pyramids	44–50	105–110	80–83.1	0.5161–0.5396	[21,22]
Au nanotriangles on glass in water	86, 100	148, 278	198.51, 339.20	0.5454, 1.3677	[23]
Au nanodisks	28–55	170–452	108–223	0.4398–0.5454	[17,24]
Ag nanoprisms	20–55	120–279	76–159	0.4662–0.6713	[17,25,26]
Au nanobowties	55–60	140–204	80–187	0.4000–0.7220	[17,27]
Au nanocups	40	210	106	0.4240	[28]
Ag nanocubes on Ag film	45–50	130–220	36.7–145	0.2097–0.5370	[29,30]
Au nanocubes on Au film	40–57	100–110	63–108	0.4200–0.6879	[31,32]
Ag nanocubes on Au film	50	280	190	0.5758	[33]
Au nanoprisms on Au film	50	180	163	0.7087	[34]
Au nanospheres on Au film	30	45	130	1.7333	[35]
Ag nanobipyramids on Ag film	32–39	66–73	35–53	0.3333–0.5048	[36]
Ga NPs on Au film	33	258	122.5	0.4210	[37]

Table S3: Spontaneous emission of QEs coupled with various plasmonic nanostructures.

**Plasmonic nanostructures**	**QEs**	**Nanostructure size [nm]**[a]	**PL enhance.**	**Ref.**
Ag nanocube on Ag film	NV centers	Nanocube $l \approx 100$	90–300	[38]
Ag nanocube on Ag film	Cy5 dye	Nanocube $l \approx 83.6$	30000 [b]	[39]
Ag nanocube on Au film	Ru dye / QDs	Nanocube $l \approx$ 75–80	65–2300	[40–42]
Ag nanocube on Au film	Monolayer WSe_2	Nanocube $l \approx$ 50–100	1700–6000	[29]
	Monolayer $MoSe_2$	Nanocube $l \approx 65$	≈6000	[43]
	InSe	Nanocube $l \approx 110$	34000 [b]	[44]
Au NP on Au film	Monolayer MoS_2	NP $d \approx 200$	350	[45]
Au nanocube on Au film	Single defects in monolayer WSe_2	Nanocube $l \approx 110$, $h \approx 90$	13	[46]
	Single defects in carbon nanotubes	Nanocube $l \approx 160$, $h \approx 30$	415	[47]
Au nanorod on Au film	Nile blue dye	Nanorod $l \approx 82.5$, $d \approx 33$	Maximum 312	[48]
Au nanopatch on Au film	Single QDs	Nanopatch $d \approx$ 200–2500, $t \approx 20$	70	[49]
Si NP on Au film	QDs	NP $d \approx$ 80–200	69 ($t \approx 5$)	[50]
Au nanobowtie on Au film	Carbon nanotubes	Nanotriangle $l \approx 250$, gap ≈10–20	mean 44, max 98	[51]
Au NP dimer on Au film	Monolayer MoS_2	NP $d \approx 140$	1350	[52]

[a] d, t, h and l represent diameter, thickness, height or depth, and length or side length, respectively.
[b] Double resonances (at excitation and emission wavelengths).

References

(1) Wu, T.; Arrivault, D.; Yan, W.; Lalanne, P. Modal analysis of electromagnetic resonators: user guide for the MAN program. *Computer Physics Communications* **2023**, *284*, 108627.

(2) Ciracì, C.; Hill, R. T.; Mock, J. J.; Urzhumov, Y.; Fernández-Domínguez, A. I.; Maier, S. A.; Pendry, J. B.; Chilkoti, A.; Smith, D. R. Probing the Ultimate Limits of Plasmonic Enhancement. *Science* **2012**, *337*, 1072–1074.

(3) Lin, X.; Fang, G.; Liu, Y.; He, Y.; Wang, L.; Dong, B. Marangoni Effect-Driven Transfer and Compression at Three-Phase Interfaces for Highly Reproducible Nanoparticle Monolayers. *The Journal of Physical Chemistry Letters* **2020**, *11*, 3573–3581.

(4) Li, B. et al. Scalable Transfer of Suspended Two-Dimensional Single Crystals. *Nano Letters* **2015**, *15*, 5089–5097.

(5) Weber, T.; Kühner, L.; Sortino, L.; Ben Mhenni, A.; Wilson, N. P.; Kühne, J.; Finley, J. J.; Maier, S. A.; Tittl, A. Intrinsic strong light-matter coupling with self-hybridized bound states in the continuum in van der Waals metasurfaces. *Nature Materials* **2023**, *22*, 970–976.

(6) Wang, S.; Li, S.; Chervy, T.; Shalabney, A.; Azzini, S.; Orgiu, E.; Hutchison, J. A.; Genet, C.; Samorì, P.; Ebbesen, T. W. Coherent Coupling of WS_2 Monolayers with Metallic Photonic Nanostructures at Room Temperature. *Nano Letters* **2016**, *16*, 4368–4374.

(7) Sang, Y.; Wang, C.-Y.; Raja, S. S.; Cheng, C.-W.; Huang, C.-T.; Chen, C.-A.; Zhang, X.-Q.; Ahn, H.; Shih, C.-K.; Lee, Y.-H.; Shi, J.; Gwo, S. Tuning of Two-Dimensional Plasmon–Exciton Coupling in Full Parameter Space: A Polaritonic Non-Hermitian System. *Nano Letters* **2021**, *21*, 2596–2602.

(8) Yu, M.-W.; Ishii, S.; Li, S.; Ku, J.-R.; Yang, J.-H.; Su, K.-L.; Taniguchi, T.; Nagao, T.; Chen, K.-P. Quantifying photoinduced carriers transport in exciton–polariton coupling of MoS_2 monolayers. *npj 2D Materials and Applications* **2021**, *5*, 47.

(9) Ding, B.; Zhang, Z.; Chen, Y.-H.; Zhang, Y.; Blaikie, R. J.; Qiu, M. Tunable Valley Polarized Plasmon-Exciton Polaritons in Two-Dimensional Semiconductors. *ACS Nano* **2019**, *13*, 1333–1341.

(10) Wang, S.; Le-Van, Q.; Vaianella, F.; Maes, B.; Eizagirre Barker, S.; Godiksen, R. H.; Curto, A. G.; Gomez Rivas, J. Limits to Strong Coupling of Excitons in Multilayer WS_2 with Collective Plasmonic Resonances. *ACS Photonics* **2019**, *6*, 286–293.

(11) Zheng, D.; Zhang, S.; Deng, Q.; Kang, M.; Nordlander, P.; Xu, H. Manipulating Coherent Plasmon–Exciton Interaction in a Single Silver Nanorod on Monolayer WSe_2. *Nano Letters* **2017**, *17*, 3809–3814.

(12) Li, C.; Lu, X.; Srivastava, A.; Storm, S. D.; Gelfand, R.; Pelton, M.; Sukharev, M.; Harutyunyan, H. Second Harmonic Generation from a Single Plasmonic Nanorod Strongly Coupled to a WSe_2 Monolayer. *Nano Letters* **2021**, *21*, 1599–1605.

(13) Wang, Y.; You, Q.; Li, Z.; Zhang, L.; Zhang, D.; Wang, P. Strong Coupling of Plasmonic Nanorods with a $MoSe_2$ Monolayer in the Near-Infrared Shortwave Region. *The Journal of Physical Chemistry C* **2024**, *128*, 5280–5287.

(14) Wen, J.; Wang, H.; Wang, W.; Deng, Z.; Zhuang, C.; Zhang, Y.; Liu, F.; She, J.; Chen, J.; Chen, H.; Deng, S.; Xu, N. Room-Temperature Strong Light–Matter Interaction with Active Control in Single Plasmonic Nanorod Coupled with Two-Dimensional Atomic Crystals. *Nano Letters* **2017**, *17*, 4689–4697.

(15) Jiang, Y.; Wang, H.; Wen, S.; Chen, H.; Deng, S. Resonance Coupling in an Individual Gold Nanorod-Monolayer WS_2 Heterostructure: Photoluminescence Enhancement with Spectral Broadening. *ACS nano* **2020**, *14*, 13841–13851.

(16) Wen, J.; Wang, H.; Chen, H.; Deng, S.; Xu, N. Room-temperature strong coupling between dipolar plasmon resonance in single gold nanorod and two-dimensional excitons in monolayer WSe_2. *Chinese Physics B* **2018**, *27*, 096101.

(17) Yan, X.; Wei, H. Strong plasmon–exciton coupling between lithographically defined single metal nanoparticles and monolayer WSe_2. *Nanoscale* **2020**, *12*, 9708–9716.

(18) Niu, Y.; Xu, H.; Wei, H. Unified Scattering and Photoluminescence Spectra for Strong Plasmon-Exciton Coupling. *Physical Review Letters* **2022**, *128*, 167402.

(19) Zhong, J.; Li, J.-Y.; Liu, J.; Xiang, Y.; Feng, H.; Liu, R.; Li, W.; Wang, X.-H. Room-Temperature Strong Coupling of Few-Exciton in a Monolayer WS_2 with Plasmon and Dispersion Deviation. *Nano Letters* **2024**, *24*, 1579–1586.

(20) Lo, T. W.; Zhang, Q.; Qiu, M.; Guo, X.; Meng, Y.; Zhu, Y.; Xiao, J. J.; Jin, W.; Leung, C. W.; Lei, D. Thermal Redistribution of Exciton Population in Monolayer Transition Metal Dichalcogenides Probed with Plasmon–Exciton Coupling Spectroscopy. *ACS Photonics* **2019**, *6*, 411–421.

(21) Stührenberg, M.; Munkhbat, B.; Baranov, D. G.; Cuadra, J.; Yankovich, A. B.; Antosiewicz, T. J.; Olsson, E.; Shegai, T. Strong Light–Matter Coupling between Plasmons in Individual Gold Bi-pyramids and Excitons in Mono- and Multilayer WSe_2. *Nano Letters* **2018**, *18*, 5938–5945.

(22) Lawless, J.; Hrelescu, C.; Elliott, C.; Peters, L.; McEvoy, N.; Bradley, A. L. Influence of Gold Nano-Bipyramid Dimensions on Strong Coupling with Excitons of Monolayer MoS_2. *ACS Applied Materials & Interfaces* **2020**, *12*, 46406–46415.

(23) Wang, M.; Krasnok, A.; Zhang, T.; Scarabelli, L.; Liu, H.; Wu, Z.; Liz-Marzán, L. M.; Terrones, M.; Alù, A.; Zheng, Y. Tunable Fano Resonance and Plasmon–Exciton Coupling in Single Au Nanotriangles on Monolayer WS_2 at Room Temperature. *Advanced Materials* **2018**, *30*, 1705779.

(24) Geisler, M.; Cui, X.; Wang, J.; Rindzevicius, T.; Gammelgaard, L.; Jessen, B. S.; Gonçalves, P. A. D.; Todisco, F.; Bøggild, P.; Boisen, A.; Wubs, M.; Mortensen, N. A.; Xiao, S.; Stenger, N. Single-Crystalline Gold Nanodisks on WS_2 Mono- and Multilayers for Strong Coupling at Room Temperature. *ACS Photonics* **2019**, *6*, 994–1001.

(25) Cuadra, J.; Baranov, D. G.; Wersäll, M.; Verre, R.; Antosiewicz, T. J.; Shegai, T. Observation of Tunable Charged Exciton Polaritons in Hybrid Monolayer WS_2-Plasmonic Nanoantenna System. *Nano Letters* **2018**, *18*, 1777–1785.

(26) Munkhbat, B.; Baranov, D. G.; Bisht, A.; Hoque, M. A.; Karpiak, B.; Dash, S. P.; Shegai, T. Electrical Control of Hybrid Monolayer Tungsten Disulfide–Plasmonic Nanoantenna Light–Matter States at Cryogenic and Room Temperatures. *ACS Nano* **2020**, *14*, 1196–1206.

(27) Yang, L. et al. Strong Light–Matter Interactions between Gap Plasmons and Two-Dimensional Excitons under Ambient Conditions in a Deterministic Way. *Nano Letters* **2022**, *22*, 2177–2186.

(28) Ai, R.; Xia, X.; Zhang, H.; Chui, K. K.; Wang, J. Orientation-Dependent Interaction between the Magnetic Plasmons in Gold Nanocups and the Excitons in WS_2 Monolayer and Multilayer. *ACS Nano* **2023**, *17*, 2356–2367.

(29) Sun, J.; Hu, H.; Zheng, D.; Zhang, D.; Deng, Q.; Zhang, S.; Xu, H. Light-Emitting Plexciton: Exploiting Plasmon–Exciton Interaction in the Intermediate Coupling Regime. *ACS Nano* **2018**, *12*, 10393–10402.

(30) Han, X.; Wang, K.; Xing, X.; Wang, M.; Lu, P. Rabi Splitting in a Plasmonic Nanocavity Coupled to a WS_2 Monolayer at Room Temperature. *ACS Photonics* **2018**, *5*, 3970–3976.

(31) Zheng, J.; Krasavin, A. V.; Yang, R.; Wang, Z.; Feng, Y.; Tang, L.; Li, L.; Guo, X.; Dai, D.; Zayats, A. V.; Tong, L.; Wang, P. Active control of excitonic strong coupling

and electroluminescence in electrically driven plasmonic nanocavities. *Science Advances* **2025**, *11*, eadt9808.

(32) Li, C.; Luo, H.; Hou, L.; Wang, Q.; Liu, K.; Gan, X.; Zhao, J.; Xiao, F. Giant Photoluminescence Enhancement of Monolayer WSe_2 Using a Plasmonic Nanocavity with On-Demand Resonance. *Nano Letters* **2024**, *24*, 5879–5885.

(33) Hou, S.; Tobing, L. Y. M.; Wang, X.; Xie, Z.; Yu, J.; Zhou, J.; Zhang, D.; Dang, C.; Coquet, P.; Tay, B. K.; Birowosuto, M. D.; Teo, E. H. T.; Wang, H. Manipulating Coherent Light–Matter Interaction: Continuous Transition between Strong Coupling and Weak Coupling in MoS_2 Monolayer Coupled with Plasmonic Nanocavities. *Advanced Optical Materials* **2019**, *7*, 1900857.

(34) Qin, J.; Chen, Y.-H.; Zhang, Z.; Zhang, Y.; Blaikie, R. J.; Ding, B.; Qiu, M. Revealing Strong Plasmon-Exciton Coupling between Nanogap Resonators and Two-Dimensional Semiconductors at Ambient Conditions. *Physical Review Letters* **2020**, *124*, 063902.

(35) Liu, X.; Yi, J.; Yang, S.; Lin, E.-C.; Zhang, Y.-J.; Zhang, P.; Li, J.-F.; Wang, Y.; Lee, Y.-H.; Tian, Z.-Q.; Zhang, X. Nonlinear valley phonon scattering under the strong coupling regime. *Nature Materials* **2021**, *20*, 1210–1215.

(36) Lu, Z.; Song, D.; Lin, C.; Zhang, H.; Zhang, S.; Xu, H. Plexciton Photoluminescence in Strongly Coupled 2D Semiconductor–Plasmonic Nanocavity Hybrid. *ACS Nano* **2025**, *19*, 5637–5648.

(37) Deng, F.; Liu, H.; Xu, L.; Lan, S.; Miroshnichenko, A. E. Strong Exciton–Plasmon Coupling in a WS_2 Monolayer on Au Film Hybrid Structures Mediated by Liquid Ga Nanoparticles. *Laser & Photonics Reviews* **2020**, *14*, 1900420.

(38) Bogdanov, S. I.; Shalaginov, M. Y.; Lagutchev, A. S.; Chiang, C.-C.; Shah, D.; Baburin, A. S.; Ryzhikov, I. A.; Rodionov, I. A.; Kildishev, A. V.; Boltasseva, A.;

Shalaev, V. M. Ultrabright Room-Temperature Sub-Nanosecond Emission from Single Nitrogen-Vacancy Centers Coupled to Nanopatch Antennas. *Nano Letters* **2018**, *18*, 4837–4844.

(39) Rose, A.; Hoang, T. B.; McGuire, F.; Mock, J. J.; Ciracì, C.; Smith, D. R.; Mikkelsen, M. H. Control of Radiative Processes Using Tunable Plasmonic Nanopatch Antennas. *Nano Letters* **2014**, *14*, 4797–4802.

(40) Akselrod, G. M.; Argyropoulos, C.; Hoang, T. B.; Ciracì, C.; Fang, C.; Huang, J.; Smith, D. R.; Mikkelsen, M. H. Probing the mechanisms of large Purcell enhancement in plasmonic nanoantennas. *Nature Photonics* **2014**, *8*, 835–840.

(41) Hoang, T. B.; Akselrod, G. M.; Argyropoulos, C.; Huang, J.; Smith, D. R.; Mikkelsen, M. H. Ultrafast spontaneous emission source using plasmonic nanoantennas. *Nature Communications* **2015**, *6*, 7788.

(42) Hoang, T. B.; Akselrod, G. M.; Mikkelsen, M. H. Ultrafast Room-Temperature Single Photon Emission from Quantum Dots Coupled to Plasmonic Nanocavities. *Nano Letters* **2016**, *16*, 270–275.

(43) Zhang, Y.; Chen, W.; Fu, T.; Sun, J.; Zhang, D.; Li, Y.; Zhang, S.; Xu, H. Simultaneous Surface-Enhanced Resonant Raman and Fluorescence Spectroscopy of Monolayer $MoSe_2$: Determination of Ultrafast Decay Rates in Nanometer Dimension. *Nano Letters* **2019**, *19*, 6284–6291.

(44) Bao, X. et al. Giant Out-of-Plane Exciton Emission Enhancement in Two-Dimensional Indium Selenide via a Plasmonic Nanocavity. *Nano Letters* **2023**, *23*, 3716–3723.

(45) Qi, X.; Lo, T. W.; Liu, D.; Feng, L.; Chen, Y.; Wu, Y.; Ren, H.; Guo, G.-C.; Lei, D.; Ren, X. Effects of gap thickness and emitter location on the photoluminescence enhancement of monolayer MoS_2 in a plasmonic nanoparticle-film coupled system. *Nanophotonics* **2020**, *9*, 2097–2105.

(46) Luo, Y.; Shepard, G. D.; Ardelean, J. V.; Rhodes, D. A.; Kim, B.; Barmak, K.; Hone, J. C.; Strauf, S. Deterministic coupling of site-controlled quantum emitters in monolayer WSe_2 to plasmonic nanocavities. *Nature Nanotechnology* **2018**, *13*, 1137–1142.

(47) Luo, Y.; He, X.; Kim, Y.; Blackburn, J. L.; Doorn, S. K.; Htoon, H.; Strauf, S. Carbon Nanotube Color Centers in Plasmonic Nanocavities: A Path to Photon Indistinguishability at Telecom Bands. *Nano Letters* **2019**, *19*, 9037–9044.

(48) Wang, H.; Lin, Y.; Ma, P.; Zhong, Y.; Liu, H. Tunable fluorescence emission of molecules with controllable positions within the metallic nanogap between gold nanorods and a gold film. *Journal of Materials Chemistry C* **2019**, *7*, 13526–13535.

(49) Dhawan, A. R.; Belacel, C.; Esparza-Villa, J. U.; Nasilowski, M.; Wang, Z.; Schwob, C.; Hugonin, J.-P.; Coolen, L.; Dubertret, B.; Senellart, P.; Maître, A. Extreme multiexciton emission from deterministically assembled single-emitter subwavelength plasmonic patch antennas. *Light: Science & Applications* **2020**, *9*, 33.

(50) Yang, G.; Niu, Y.; Wei, H.; Bai, B.; Sun, H.-B. Greatly amplified spontaneous emission of colloidal quantum dots mediated by a dielectric-plasmonic hybrid nanoantenna. *Nanophotonics* **2019**, *8*, 2313–2319.

(51) Luo, Y.; Ahmadi, E. D.; Shayan, K.; Ma, Y.; Mistry, K. S.; Zhang, C.; Hone, J.; Blackburn, J. L.; Strauf, S. Purcell-enhanced quantum yield from carbon nanotube excitons coupled to plasmonic nanocavities. *Nature Communications* **2017**, *8*, 1413.

(52) Cao, S.; Hou, L.; Wang, Q.; Li, C.; Yu, W.; Gan, X.; Liu, K.; Premaratne, M.; Xiao, F.; Zhao, J. Augmenting photoluminescence of monolayer MoS2 using high order modes in a metal dimer-on-film nanocavity. *Photonics Research* **2021**, *9*, 501.
